# Four-dimensional noncontrast-enhanced MR angiography at ultrahigh field

**DOI:** 10.1186/1532-429X-15-S1-W11

**Published:** 2013-01-30

**Authors:** GJ Metzger, S Schmitter, X Li, P Van de Moortele, P Schmitt, X Bi

**Affiliations:** 1 Center for Magnetic Resonance Research, University of Minnesota, Minneapolis, MN, USA; 2 MR Application & Workflow Development, Siemens AG, Erlangen, Germany; 3 Cardiovascular MR R&D, Siemens Healthcare, Chicago, IL, USA

## Background

Arterial spin labeled (ASL) 4D MRA has been shown to be a valuable method for simultaneously assessing anatomic structure and dynamic filling of cerebral arteries. Beyond improved SNR and parallel imaging performance, performing these studies at ultrahigh field will benefit from longer T1s resulting in more persistent labeling allowing for improved visualization of distal vessels, especially in conditions of reduced flow.

## Methods

**Figure 1:**
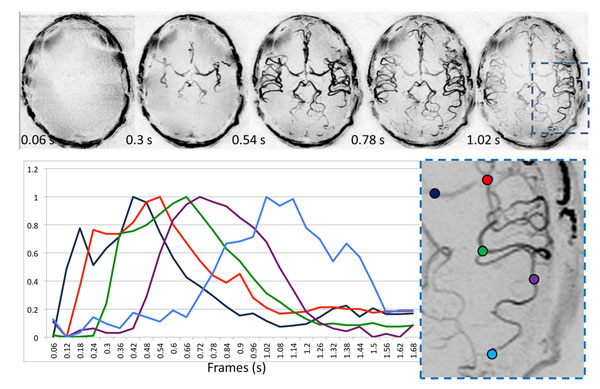
Volumetric MIPs at every 4th frame up to 1.02 s post labeling (upper row). Normalized signal intensity over 2 RR intervals at 5 locations over all 28 frames encompassing 1.68 seconds after labeling (lower row). The zoomed region on which the ROIs are placed is demarcated in the last frame of the upper row..

Studies were performed on Siemens 7T whole body MRI scanner with a 16-channel transceiver TEM stripline array head coil driven by a series of 16, 1 kW amplifiers (CPC, Pittsburgh, PA). Non-contrast enhanced 4D ASL MRA was performed using an ECG-triggered, segmented, 3D acquisition using signal targeting with alternating radiofrequency (STAR) for spin labeling. Label and control volumes were acquired with magnetization preparation immediately after the ECG R-wave and before the segmented readout used to generate the time resolved frames (Fig. 1). Background saturation over the imaging volume was applied for both label and control volumes. Data was acquired with 26 frames over 1700 ms (two R-R intervals) with a temporal resolution of ~60 ms and an acquisition time of 12:23.

To address the challenges of B1+ homogeneity and efficiency at 7T, B1+ shimming was performed based on a small flip angle, calibration scan. Two B1+ shimming strategies were compared; 1) a static approach which used a CP-like mode over the entire brain for all RF pulses in the sequence, and 2) a dynamic approach which used a separate B1+ shim optimized for efficiency for the IR labeling pulse (Shim 1) and the CP-like mode for excitation and background suppression (Shim 2) (Fig. 1).

## Results

**Figure 2:**
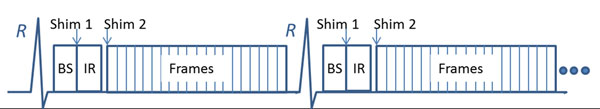
4D ASL MRA sequence. The label volume is acquired with an inversion labeling (IR) pulse on and the control volume with the IR pulse off. Background suppression (BS) is applied in the imaging volume for label and control volume acquisitions. The locations for the dynamically applied B1+ shim solutions are shown by the labels “Shim 1” and “Shim 2”.

The CP-like mode provides reasonable transmit efficiency and homogeneity over the head and is a sensible solution for RF pulses applied within the imaging volume. At the location of the labeling volume however, B1+ is appreciably weaker and thus benefits from its own shim focusing on optimized efficiency. By using an efficient solution optimized specifically on the labeling volume for the IR pulse and the CP-like mode for excitation and background suppression, far superior images were obtained. Higher labeling efficiency in the dynamically applied B1+ shimming strategy resulted in higher contrast between the vessels and surrounding tissue. With this acquisition strategy and the increased T1s of blood at 7T, dynamics of distal cerebral vessels were nicely characterized out to 1.56 s post labeling (Fig. 2).

## Conclusions

A dynamically applied transmit B1 (B1+) shimming strategy was employed to accommodate limitations on peak B1+, B1+ homogeneity and SAR for four-dimensional non-contrast enhanced angiography at 7T. Initial results showing signal persistence in distal arteries is made possible by the increased labeling efficiency of the dynamic B1+ shimming approach as well as the increased T1s of blood at ultrahigh field.

## Funding

Funding Provided by P41 EB015894, S10 RR26783 and WM KECK Foundation.

